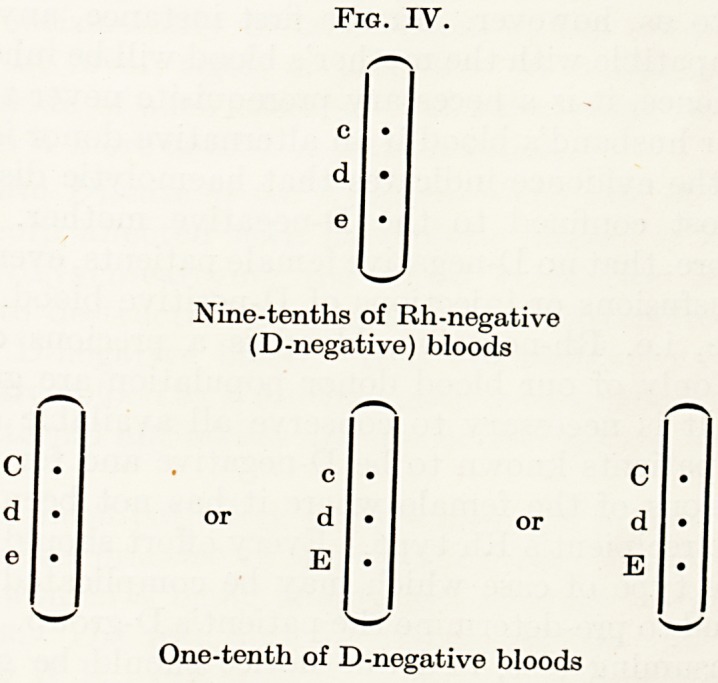# The Rhesus Factors—I: Serology

**Published:** 1947

**Authors:** Geoffrey H. Tovey

**Affiliations:** Regional Transfusion Officer, S.W. Region. Special Lecturer in Hæmatology, University of Bristol


### THE RHESUS FACTORS—I

### *Serology

BY

Dr. Geoffrey H. Tovey, M.D.

*Regional Transfusion Officer, S.W. Region.*

*Special Lecturer in Hæmatology, University of Bristol.*

Human red blood corpuscles contain many antigens besides the A and B factors, those of greatest clinical importance being the Rh factors. The first of these factors was discovered by Landsteiner and Wiener (1940), and was so called because a similar factor is present in the corpuscles of the Rhesus monkey. This factor is demonstrable in the corpuscles of 85 per cent. of Europeans and white Americans, who are said to be Rh-positive. The remaining 15 per cent. lacking this factor are known as Rh-negative.

When the agglutinogens A or B are absent from the corpuscles the corresponding antibodies, anti-A or anti-B, are present in the plasma (see Fig. I). The Rh factor differs from the A and B factors in that no *natural* Rh antibodies, anti-Rh, are found in the plasma of Rh-negative blood. Such antibodies may appear, however, as the result of immunization.

Fig. I. The Four Blood Groups.

| Corpuscular antigens (agglutinogens) | Plasma (agglutinins) |
| --- | --- |
| AB | None |
| A | anti-B |
| B | anti-A |
| O | anti-A and anti-B |

*Immunization by the Rh antigen* may occur in three ways : By the subcutaneous or intramuscular injection of Rh-positive blood into a Rh-negative person, e.g. in measles prophylaxis; by the transfusion of Rh-positive blood to a Rh-negative recipient ; and by a Rh-negative mother carrying a Rh-positive foetus. In each instance immunization will manifest itself by the appearance of Rh

* A paper read to the Society on Wednesday, May 14th, 1947.

antibodies in the plasma, which is of clinical importance in blood transfusion therapy and in pregnancy.

*Blood Transfusion.*—The giving of Rh-positive blood to a Rh-negative recipient with preformed Rh antibodies in the plasma will result in the haemolysis of the transfused corpuscles. If the Rh-positive corpuscles are broken down rapidly, a dangerous, and sometimes a fatal, reaction occurs. If they are haemolysed more slowly, the patient may escape a severe reaction, but will fail to derive the maximum benefit from the transfusion. Moreover, the Rh antibodies will be considerably increased in potency, so that unless due precautions are taken, a subsequent transfusion may prove fatal. Such recipients will tolerate only blood that does not contain the offending Rh antigen, i.e. Rh-negative blood.

*Pregnancy.*—In ten per cent. of all pregnancies the foetus is Rh-positive and its mother Rh-negative. In certain instances the Rh-negative mother will react to the presence of the Rh-positive foetus as if she had received a transfusion of Rh-positive blood, and Rh antibodies will appear in her plasma. The placental barrier is freely permeable to these antibodies (Tovey, 1945), which pass over into the foetal circulation and there react with the Rh factor in the baby's corpuscles and tissue cells. As a result of the interaction of Rh antigen and antibody *in utero* the baby suffers from a condition now known as haemolytic disease of the newborn (erythroblastosis foetalis). Haemolytic disease of the newborn complicates one in every two-hundred to two-hundred-and-fifty pregnancies, and is responsible for more deaths than any other inherited condition, perhaps more than all other inherited conditions together (Haldane, 1943).

### SUB-DIVISIONS OF THE RH FACTOR

Serological investigations in cases of haemolytic disease have shown that in 92 per cent. of cases the mother is Rh-negative and her baby Rh-positive, but that the remaining 8 per cent. of cases occur in Rh-positive women who have been immunized by a related antigen present in the foetal corpuscles but lacking from their own blood. These cases are explained by the fact that all human corpuscles contain, instead of a simple Rh factor, a minimum of three and a maximum of six Rh factors. This fact will become clearer if we consider briefly the chromosomes responsible for the Rh content of our red blood corpuscles and tissue cells.

It is thought (Fisher, quoted by Race, 1944) that the Rh factors are handed on from parent to child by three closely-linked genes on the chromosome pair. At each of three loci is found one or other of two alternative genes, which, to bring them into line with the ABO factors, are designated C, c, D, d, E and e. The gene indicated by each of these six letters determines the presence of a distinctive antigen in the red cells.

FIG. II.

Diagram showing the alternative occupants (allelomorphs) of the three closely linked loci on the chromosome bearing the Rh factors

We may thus inherit a minimum of three and a maximum of six distinct Rh factors from our parents, depending upon their Rh types.

FIG. III.

Chromosome from mother — Chromosome from father

Rh content of red cell (phenotype):
CDe
Genotype: CDe/CDe

Chromosome from mother — Chromosome from father

Rh content of red cell (phenotype);
CcDdEe
Genotype: CDe/cdE

The small letters denote alternative factors—not recessive antigens. All are of equal dominance as regards inheritance; fortunately, however, not all are of equal clinical importance. The factor D is the most potent of the six antigens and is responsible for the great majority of Rh reactions. It is the factor first to have been discovered and is the factor allied to that present in the corpuscles of the Rhesus monkey. When we refer loosely to "Rh-positive" and "Rh-negative," therefore, we really mean "D-positive" and "D-negative."

Nevertheless, the other Rh antigens may, and do, on occasion, stimulate the formation of antibodies (anti-C, anti-E, anti-c, etc.).

Thus, whilst 92 per cent. of cases of haemolytic disease of the newborn are due to a D-negative mother forming anti-D against her foetus, the remaining 8 per cent. of affected foetuses are carried by D-positive women who are sensitized by a C, c, E, e or d antigen present in the foetal corpuscles but absent from the mother's blood. This has an important clinical bearing for the following reason. When the antigen D is absent from the locus Dd on the chromosome the locus must be occupied by the antigen d. The gene complex containing d nearly always contains the combination cde (see Fig. IV).

FIG. IV.

Nine-tenths of Rh-negative (D-negative) bloods

One-tenth of D-negative bloods

Nearly all bloods classed as "Rh-negative" contain, therefore, three antigens, c, d and e. This must be borne in mind when transfusing Rh-negative blood, because should the recipient's plasma contain anti-c, anti-d, or anti-e, the "Rh-negative" corpuscles would be incompatible.

### PRACTICAL APPLICATIONS IN TRANSFUSION THERAPY AND IN HAEMOLYTIC DISEASE OF THE NEWBORN.

*Blood Transfusions.*—In theory, blood for transfusion should be of the same Rh phenotype as that of the recipient. In practice, a compromise has to be reached. There is, at present, no simple method of Rh grouping; Rh anti-sera other than anti-D are scarce: Rh grouping and the exclusion of Rh incompatibility may take one to two hours and should be undertaken by experienced workers only; blood of a particular Rh type may not be available in any quantity. Rh antibodies, on the other hand, are immune bodies and no persons having natural Rh antibodies have been found. At a first transfusion in a male, therefore, the Rh type of the recipient need not be taken into consideration.

A first transfusion in a female presents a different problem. Should the recipient be of child-bearing or pre-child-bearing years we must ever keep in mind the fact that the transfusion may sensitize her towards an antigen which may later be borne by the corpuscles of her foetus. In such circumstances the foetus is almost invariably affected with haemolytic disease in its severest forms (Levine and Waller, 1946).

Ideally, therefore, until they have reached the menopause females should be given blood of their own Rh phenotype only. This is fraught with obvious difficulties. Two essentially practical measures are available to us, however. In the first instance, any foetal Rh antigens incompatible with the mother's blood will be inherited from the father. Hence, it is a necessary prerequisite never to transfuse a wife with her husband's blood if an alternative donor is available. Secondly, all the evidence indicates that haemolytic disease of the foetus is almost confined to the D-negative mother. We must ensure, therefore, that no D-negative female patients, even as infants, are given transfusions or injections of D-positive blood.

D-negative, i.e. Rh-negative, blood is a precious commodity. Six per cent. only of our blood donor population are group O and D-negative. It is necessary to conserve all available supplies for those female patients known to be D-negative and for those emergency tranfusions of the female where it has not been possible to determine the recipient's Rh type. Every effort should be made to anticipate the type of case which may be complicated by a blood transfusion and to pre-determine the patient's D-group. D-grouping is a time-consuming test, and due notice should be given to the laboratory in all cases of planned transfusion.

When a woman who has borne children requires a transfusion, enquiry should be made whether her children have shown signs suggestive of haemolytic disease, i.e. were jaundiced or stillborn. Mothers giving this history may have potent Rh antibodies in their circulation and may react fatally if given blood incompatible with those antibodies. In 92 per cent. the antibodies will be anti-D, and it will be safe to give D-negative blood. In some of the remaining 8 per cent. the antibodies will be active against D-negative blood. In Bristol, to date, we have encountered five such cases.

The blood of women who have given birth to children with haemolytic disease should be investigated to determine which type of anti-body is present, so that should a transfusion be required subsequently, blood of the correct type will be given. Where this has not been determined, and in emergency, such women should be given saline or plasma until matched blood can be obtained.

The presence of Rh antibodies in the circulation may also complicate transfusion when a recipient has been transfused previously. Such a complication is, again, almost confined to the D-negative recipient. Recent investigations (Diamond, 1947) indicate that

50 per cent. of D-negative recipients will react to a transfusion of D-positive blood by the production of antibodies. Males, and females, therefore, receiving other than a first transfusion should be D-grouped before a subsequent transfusion is given. Saline, plasma, or D-negative blood, may be given at a repeat transfusion in cases which have not been Rh typed.

More rarely, a D-positive recipient will form immune antibodies following a transfusion. In some instances these antibodies will be active against D-negative blood. Indication may be given to the clinician that the patient is forming immune antibodies by a failure to respond satisfactorily to transfusion, or the complication of the transfusion by fever, rigor, or jaundice. A complete investigation should be made of such cases so that subsequent transfusion does not result in a more severe or fatal reaction.

*Haemolytic Disease of the Newborn.*—The aim of transfusion of the newly-born affected with haemolytic disease is to provide the baby with oxygen-carrying red blood corpuscles which are not susceptible to destruction by the maternal antibodies. Most cases will require D-negative blood. It is not satisfactory, however, to give D-negative blood as a routine, until investigation has confirmed that the maternal antibodies are not active against the D-negative corpuscles.

Since Rh incompatibility complicates one in every 200 to 250 pregnancies, the practice of determining the Rh group (D-group) of pregnant women is being undertaken at many of the larger ante-natal centres. The test may be carried out on the same sample submitted for W.R. examination. This practice has been introduced with two objectives. It is hoped that, with early diagnosis and treatment, the morbidity rate and mortality rate of haemolytic disease of the newborn will be lowered, and furthermore, that the D-group of the mother requiring transfusion during or after the pregnancy being known will both assist in the conservation of D-negative blood and will minimize the risks of sensitizing women to the D antigen. The extent to which routine testing can be developed is in part limited by the availability of stocks of anti-Rh grouping sera. Satisfactory Rh anti-sera are obtainable, only, from human sources, and obstetricians can assist greatly by persuading mothers with potent Rh antibodies to donate blood when the antibodies are at a maximal titre.

REFERENCES.

Diamond, L. K. Personal communication, 1947.

Haldane, J. B. S. *Ann. Eugenics,* 11 : 333, Dec., 1943.

Landsteiner, K., and Wiener, A. S. *J. Exper. Med.*, 74 : 309, Oct., 1941.

Levine, P., and Waller, R. K. *J. Haemat.*, 1 : 143, March, 1946.

Race, R. R. *Nature,* 153 : 771, June, 1944.

Tovey, G. H. *J. Path. & Bact.*, 57 : 295, July, 1945.

## Figures and Tables

**Fig. I. f1:**
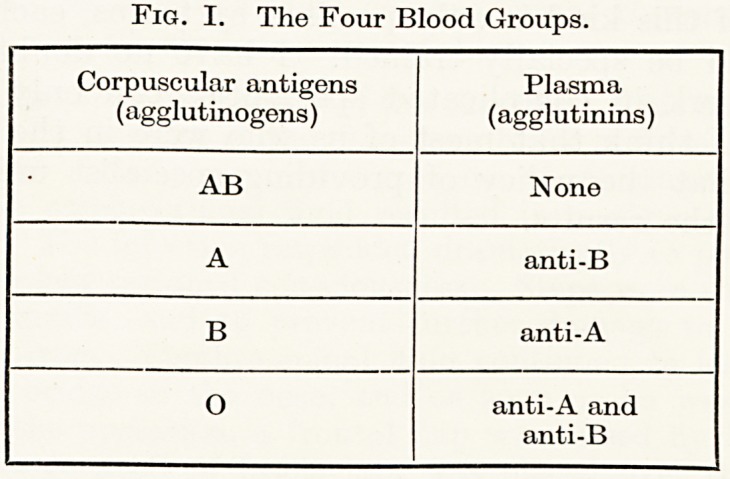


**Fig. II. f2:**
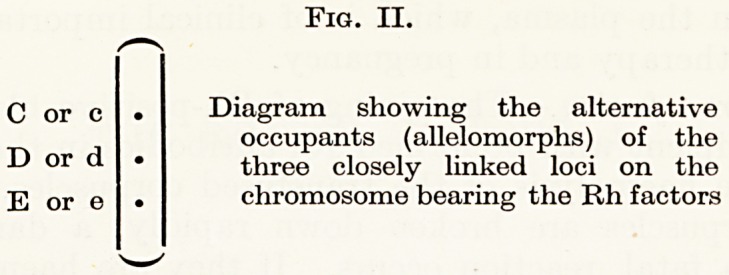


**Fig. III. f3:**
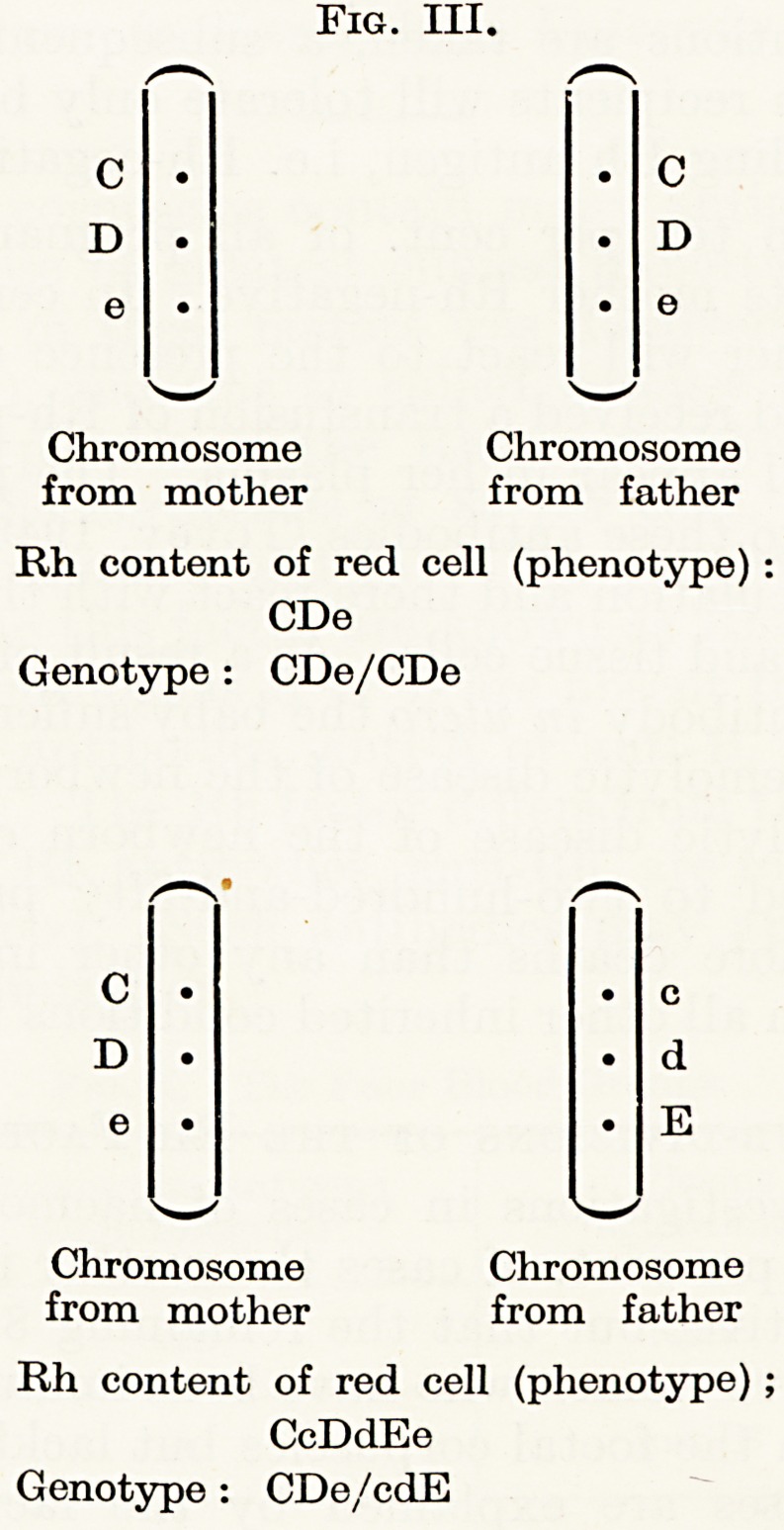


**Fig. IV. f4:**